# The Optimal Distribution of Surgery in Low- and Middle-Income Countries: A Proposed Matrix for Determining Country-Level Organization of Surgical Services – A Response to the Recent Commentaries

**DOI:** 10.34172/ijhpm.2020.168

**Published:** 2020-09-01

**Authors:** Katherine R. Iverson, Emma Svensson, Kristin Sonderman, Ernest J. Barthélemy, Isabelle Citron, Kerry A. Vaughan, Brittany L. Powell, John G. Meara, Mark G. Shrime

**Affiliations:** ^1^Program in Global Surgery and Social Change, Harvard Medical School, Boston, MA, USA.; ^2^General Surgery Department, University of California Davis Medical Center, Sacramento, CA, USA.; ^3^Lund University, Lund, Sweden.; ^4^Brigham and Women’s Hospital, Boston, MA, USA.; ^5^Icahn School of Medicine at Mount Sinai, New York City, NY, USA.; ^6^University of Pennsylvania, Philadelphia, PA, USA.; ^7^Stanford University School of Medicine, Stanford, CA, USA.; ^8^Department of Plastic and Oral Surgery, Boston Children’s Hospital, Boston, MA, USA.; ^9^Massachusetts Eye and Ear Infirmary, Boston, MA, USA.

 The insightful responses by Dr. Roder-DeWan, Dr. Henry, and Dr. Kreindler have added great richness to the ongoing conversation about the best way to organize surgical services in low- and middle-income countries. The authors keenly point out where evidence is strong, the gaps in our knowledge, and how these can be addressed to create better guidelines for the future.

 Balancing the at times competing needs of logistical access to care and quality of care remains an ongoing debate. The most robust evidence presented in our review is for the decentralization of obstetric services – ensuring emergency surgical care inclusive of cesarean sections are available closest to those who need it at either the primary or district hospital level.^1^ A caveat related to this suggestion is expounded on by Dr. Roder-DeWan who points out the need to ensure high quality in comprehensive emergency obstetric and newborn care services, which is most likely to be achieved at district hospitals as opposed to smaller community hospitals.^2-4^ Incorporating studies from high-income countries could help address this question of quality and enrich the evidence presented from low- and middle-income countries in our review, as Dr. Kriendler and Dr. Henry point out.^5,6^ This is particularly relevant for trauma care where there is strong evidence for regionalization from high-income countries, in particular the United States.^7-9^ This recommendation has recently been expanded to emergency general surgery patients, with recent studies suggesting a similar regionalized care model be extended to these cases.^10,11^ A hub-and-spoke model, as pointed about by Dr. Henry, may be best to interconnect these different facilities and levels of care for urgent surgical cases.^6^ However, a robust transportation, transfer, and referral system is required in order for those with the most severe injuries, or those who require more skilled surgical intervention, to receive treatment in a timely manner.^12^Additionally, the need to identify when a birth, trauma, or surgical condition requires a higher level of care may not always be readily apparent, thus not allowing for adequate time and consideration for transfer. Systems-level thinking looking closely at the population requiring care, the process to get to those services, and the capacity to provide surgery at these facilities will be instrumental in ensuring success of this distribution scheme.^13^ These context-specific considerations are particularly vital for implementation of these recommendations.

 Our original framework espoused a consideration of three domains of surgical care in order to guide the targeted hospital level: acuity, complexity, and volume. Improvements to these guidelines have been proposed to increase their granularity, with Dr. Henry expanding the framework to 8 unique combinations of these domains.^6^ A further step would be to map each procedure within an essential surgical package to each of these 8 levels. This could include the 44 procedures in the Disease Control Priorities, but most useful would be to use a country’s specific package of surgical procedures and consider each surgery along these areas.^14^ Additionally, specific numeric values could be assigned to each of these categories within a particular context. For example, expected yearly surgical volumes of each procedure should ideally be available from country-level or regional estimates. This would not only include the current volume, but the surgical volume required to address the unmet surgical need. A ranking system for acuity could incorporate the time from identified pathology to required surgical intervention, with emergency surgery requiring 2-hour access in the case of the Bellwether procedures of cesarean section, laparotomy, or open fracture reduction.^15^ Longer time periods would be considered for cleft lip (6 months) or palate (18 months), obstetric fistula (1 year), or oncologic diagnoses.^16^Complexity is more nuanced to quantify, but could include surrogates such as cost, resource-intensiveness (material and human), or number of specialties required for comprehensive treatment, ultimately resulting in a ranking system of the listed surgeries along this domain. For example, oncologic surgery or congenital repairs would rank as more complex with higher costs, larger number of resources, and multidisciplinary teams required versus hernia repair or hydrocelectomy requiring lower costs, less resources, and not as many health professionals. Additionally, more explicitly stating the desired hospital designation for each surgery, from primary to tertiary level, would need to be defined at the country level based on each unique organizational structure.^17^

 In the end, a matrix combining the package of procedures, quantifications of volume, acuity, and complexity, and targeted country-specific hospital levels would result. An example of this is provided in Table. While the recommendations set forth in the original review and in Dr. Henry’s paper serve as general guidelines, the specifics presented here demonstrate an exercise which would be useful in a national surgical, obstetric, and anesthesia planning strategy.^18^Considerations of the resources available at each hospital facility should be carefully considered as well as the degree of burden of these surgical diseases. This will allow for country-specific surgical needs to be matched with the underlying context of each hospital and provide targets for the build-up of surgical capacity. The best way forward is to incorporate these recommendations into government-led policy-making explicit surgical priorities. This should be accompanied by a clear monitoring and evaluation strategy to evaluate patient outcomes from the proposed organization and metrics to determine if the surgical needs of the population are being met. Future research evaluating policy implementation at this level will further the discussion regarding the optimal distribution of surgical care in these settings.^19^

**Table:** Matrix for Surgical Services Organizational Planning

**Surgery**	**Country**	**Volume (Procedures Per Year Required Per 100 000 Population)**	**Acuity (Optimal Time From Diagnosis to Surgery)**	Complexity** (Cost of Treatment in USD Per DALY Averted ^20^ **)**	Target Hospital Level*** ^14^
Elective inguinal hernia	Ghana	300^21^	1-3 months	$13^22^	District hospital (primary)
Complex trauma*	Cambodia	420^23,24^	2 hours	$77^25^	Provincial hospital (secondary)
Obstetric fistula	Uganda	5-15^26,27^	1 year	$54^23^	National referral hospital (tertiary)

Abbreviations: USD, United States Dollar; DALY, disability-adjusted life year. ^*^Complex trauma is defined by injury requiring procedural intervention as defined by Higashi et al.^24^ ^**^Cost per DALY averted was used as a surrogate for complexity to facilitate comparison between different countries, but total cost of providing each procedure, and not per DALY averted, may be more appropriate to compare procedures within the same country. ^***^The country’s specific target hospital level is listed with parentheses showing primary, secondary, or tertiary level based on Disease Control Priorities Volume 3 designations.

## Ethical issues

 Not applicable.

## Competing interests

 Authors declare that they have no competing interests.

## Authors’ contributions

 KRI drafted the correspondence. All authors contributed to critical revisions and have seen and approved the final manuscript.

## Authors’ affiliations

^1^Program in Global Surgery and Social Change, Harvard Medical School, Boston, MA, USA. ^2^General Surgery Department, University of California Davis Medical Center, Sacramento, CA, USA. ^3^Lund University, Lund, Sweden. ^4^Brigham and Women’s Hospital, Boston, MA, USA. ^5^Icahn School of Medicine at Mount Sinai, New York City, NY, USA. ^6^University of Pennsylvania, Philadelphia, PA, USA. ^7^Stanford University School of Medicine, Stanford, CA, USA. ^8^Department of Plastic and Oral Surgery, Boston Children’s Hospital, Boston, MA, USA. ^9^Massachusetts Eye and Ear Infirmary, Boston, MA, USA.
